# Functional convergence amid taxonomic variability in gut microbiome–immune checkpoint inhibitor research: a bibliometric and mechanistic synthesis

**DOI:** 10.3389/fimmu.2026.1883259

**Published:** 2026-07-14

**Authors:** Yousef N. Alanazi

**Affiliations:** Department of Medical Laboratory Technology, College of Applied Medical Sciences, Northern Border University, Arar, Saudi Arabia

**Keywords:** bibliometric analysis, biomarkers, fecal microbiota transplantation, functional convergence, gut microbiome, immune checkpoint inhibitors, microbial metabolites, tumor microenvironment

## Abstract

Immune checkpoint inhibitors (ICIs) have transformed cancer therapy, yet clinical responses remain highly variable, and microbiome-associated findings lack reproducibility across studies. Increasing evidence implicates the gut microbiome in modulating ICI efficacy; however, findings remain inconsistent at the taxonomic level, raising the possibility that functionally convergent immunological mechanisms may underlie this apparent variability. To address this, a critical synthesis was conducted, integrating bibliometric mapping of publications indexed in the Web of Science Core Collection (2013–2025; n = 2,195) with a secondary analysis of ClinicalTrials.gov to evaluate interventional activity. Bibliometric approaches assessed scientific production, thematic evolution, and co-citation structure, complemented by a cross-cohort functional integration of representative clinical and preclinical studies to evaluate whether microbiome–ICI interactions converge on shared immunological pathways despite divergent taxonomic signatures. Publication output increased steadily, with a marked translational surge following landmark clinical studies in 2018 and a peak in trial initiation in 2021. Thematic analyses revealed a shift from mechanistic and tumor-centered research toward clinically oriented and intervention-driven themes, including microbiome modulation, microbial metabolites, and the tumor microenvironment. Although individual response-associated taxa differed substantially across independent cohorts, qualitative functional integration supported a model of convergence in immunomodulatory pathways involving short-chain fatty acid production, dendritic cell activation, and CD8^+^ T-cell priming. Collectively, these findings suggest that apparent taxonomic inconsistencies across microbiome–ICI studies may reflect underlying functional convergence rather than biological contradiction, supporting a shift toward function-based frameworks for biomarker discovery and microbiome-directed immunomodulation.

## 1 Introduction

A long-standing objective of cancer research is to activate the immune system to eradicate cancer cells and generate clinically significant responses. Immune checkpoint inhibitors (ICIs) are among the most promising treatment strategies for stimulating antitumor immunity. Cytotoxic T-lymphocyte-associated protein 4 (CTLA-4), programmed death-1 (PD-1), and its ligand PD-L1 are key immune checkpoints targeted by ICIs and have transformed cancer therapy over the past decade. Therapies targeting PD-1, PD-L1, and CTLA-4 have yielded sustained responses in metastatic melanoma (1), non-small cell lung cancer (NSCLC) (2), urothelial carcinoma (3), and gastric adenocarcinoma (4). Despite their success, response rates are markedly inconsistent and immune-related side effects occur frequently. For example, pneumonitis and thyroiditis have been reported more frequently with anti–PD-1 therapy, whereas colitis and hypophysitis appear to be more prevalent with anti–CTLA-4 medication (5–8). This heterogeneity in therapeutic outcomes has prompted extensive investigation into the biological and environmental factors that influence treatment success.

Among the biological and environmental factors influencing ICI efficacy, the gut microbiome has recently gained considerable attention as a critical regulator of antitumor immunity. The gut microbial community is now recognized as an active component of the host immune system that influences treatment response and systemic inflammation. Research across multiple tumor types has indicated that the gut microbiota composition may contribute to the efficacy of ICIs (9–11). Numerous preclinical and clinical studies have demonstrated a correlation between the gut microbiome and the effectiveness of ICIs (10, 12). Seminal studies published between 2015 and 2018 first demonstrated that oral administration of Bifidobacterium alone achieved tumor control comparable to anti–PD-L1 checkpoint blockade (12) and that Akkermansia muciniphila supplementation restored PD-1 blockade efficacy in an interleukin-12–dependent manner following fecal microbiota transfer from non-responders (11). Building on these early findings, subsequent studies reported additional bacterial species linked to responses to ICIs (13). Together, these observations suggest that gut microbiota plays an important role in shaping the effectiveness of immune checkpoint therapy.

Although the gut microbiome has recently attracted increasing attention as a modulator of ICIs, significant controversy remains in this field. Several studies have reported that diverse bacterial taxa can modulate the efficacy of ICIs in treating cancer (14–19); however, no clear consensus has been reached on the specific bacterial species that can be used in combination with ICIs. Additionally, direct comparisons are challenging due to methodological variations in donor selection, sequencing approaches, and endpoints. Although therapeutic strategies, including fecal microbiota transplantation (FMT), have demonstrated potential in preliminary trials (20, 21), substantial concerns persist regarding donor-recipient compatibility, long-term response, and infection transmission. Research in this area spans multiple disciplines, including microbiology, immunology, and oncology, and each field approaches the topic from a different perspective using its own methods. Consequently, findings are often difficult to integrate, making formation of a clear and consistent understanding of the evidence challenging. Importantly, the reproducibility of microbiome-associated signatures across independent clinical cohorts remains limited, with considerable variability in reported taxa. This raises the possibility that functionally convergent microbial and immunological mechanisms may underlie taxonomic variability across studies.

Bibliometric analysis has been widely employed to examine developmental trends and primary findings of scientific research through quantitative analysis of prior publications. This approach enables researchers to identify research hotspots, pivotal publications, and thematic evolution over time. Previous reviews of microbiome–ICI research have largely been narrative or focused on specific taxa, mechanisms, or tumor types, and the few bibliometric analyses available have remained descriptive, mapping publication trends without integrating biological interpretation. To date, no comprehensive synthesis has systematically combined large-scale bibliometric mapping with translational trial activity and a cross-cohort functional framework capable of reconciling taxonomic variability with shared immunological mechanisms.

Although numerous reviews have summarized microbiome associations with immune checkpoint inhibitor response, most remain taxon-centered and descriptive, focusing on which microbes are differentially abundant in responders versus non-responders. In contrast, the present synthesis focuses on the recurrent convergence of microbial immune-modulatory functions across heterogeneous cohorts, integrating bibliometric structure, cross-cohort functional interpretation, and interventional trial activity into a unified framework. To our knowledge, this is the first review to apply this integrative approach to the microbiome–ICI axis. This study addresses that gap by integrating bibliometric mapping with a cross-cohort functional interpretation of representative clinical and preclinical evidence. Specifically, this work contextualizes research evolution across 2013–2025, evaluates the consistency of microbiome-associated signatures across independent studies, and interprets these observations within a functional immunological framework. By combining structural and biological perspectives, this synthesis seeks to reconcile taxonomic variability with underlying biological mechanisms and to inform the development of function-based biomarkers and microbiome-directed immunotherapy strategies.

## 2 Materials and methods

This study was designed as an integrative bibliometric review combined with a structured qualitative cross-cohort functional synthesis. Two complementary analytical layers were applied. First, bibliometric mapping was performed to characterize publication trends, intellectual structure, thematic evolution, and translational development within microbiome–ICI research. Second, a predefined qualitative functional synthesis was conducted to biologically interpret representative clinical and preclinical evidence by comparing reported microbial signatures, associated metabolic pathways, and immunological mechanisms across studies. The functional integration was not intended as a quantitative meta-analysis, but as a structured comparative framework to evaluate whether heterogeneous taxonomic findings converge on shared biological mechanisms.

### 2.1 Search strategy

A bibliometric search was conducted on September 27, 2025, using the Web of Science Core Collection (WoSCC) to retrieve publications related to the gut microbiome and ICIs. Web of Science Core Collection was selected as the sole database because it provides standardized bibliographic records and cited-reference data that are well suited for bibliometric mapping, co-citation analysis, and network-based science mapping (22, 23). Although additional databases such as Scopus, PubMed, or Embase could increase retrieval breadth, WoSCC was prioritized to ensure consistency of citation metadata and compatibility with the bibliometric workflow used in this study. A topic search was performed, and full records and cited references were exported in plain text format. The search string used was as follows:

(gut microbiome OR gut microbiota OR intestinal microbiota OR intestinal microbiome OR fecal microbiota OR fecal microbiome OR gut bacteria OR gut flora OR intestinal flora)
AND
(immune checkpoint inhibitor OR ICI OR immune checkpoint blockade OR immune checkpoint therapy OR immune checkpoint inhibitors OR PD-1 OR PD1 OR PD-L1 OR PDL1 OR CTLA-4 OR CTLA4 OR pembrolizumab OR nivolumab OR ipilimumab OR atezolizumab OR avelumab OR durvalumab OR cemiplimab OR toripalimab)

### 2.2 ClinicalTrials.gov search

To evaluate the clinical maturity and translational momentum of this field, a secondary search was performed using ClinicalTrials.gov. Interventional trials were identified using the terms “microbiome” or “fecal microbiota transplant” in combination with “immune checkpoint inhibitors” or specific ICI agents (e.g., anti-PD-1). The search covered trials initiated between March 19, 2018 (following the publication of landmark human cohort studies) and September 27, 2025, and was conducted on September 27, 2025.

### 2.3 Study selection and data collection

The WoSCC search was restricted to English-language publications from 2013 to 2025. No citation-based restrictions were applied, and a full-dataset approach was used to ensure the coverage of both foundational and emerging studies in this rapidly expanding multidisciplinary field. No additional exclusion criteria were applied beyond duplicate removal, as the objective was to capture the full research landscape. A total of 2,195 records were retrieved from the WoSCC and exported with complete bibliographic information (titles, abstracts, author keywords, authors, affiliations, and references) for subsequent bibliometric analyses.

### 2.4 Bibliometric analysis

Bibliometric analyses were primarily conducted using the bibliometrix R package (24) through its Biblioshiny interface. VOSviewer (25) was used for network visualization, including keyword co-occurrence networks and overlay visualization. Reference co-citation analysis, thematic evolution, and strategic thematic mapping were performed within bibliometrix. Microsoft Excel was used to calculate and visualize annual scientific production and integrate publication trends with clinical trial registrations using a dual-axis timeline. Key milestones were annotated on the combined timeline to contextualize the observed clinical pivots and translational surges.

### 2.5 Cross-cohort functional integration

To provide a biological interpretation of the bibliometric findings, a cross-cohort functional integration was performed by synthesizing taxonomic, pathway-level, and metabolic evidence from representative clinical and preclinical studies of microbiome–ICI interactions. Studies were selected using predefined criteria: they had to report differentially abundant taxa associated with ICI response, provide statistical support for those associations (effect direction, p-value, or FDR), and define responder and non-responder groups clearly. Landmark human cohort studies and key preclinical mechanistic studies were prioritized because they were foundational to the field and provided sufficient taxonomic and functional detail for cross-study comparison. Studies without comparable response definitions or without taxon-level data were excluded. Evidence levels were assigned based on study type: Level 2, preclinical *in vivo* studies; Level 3, human observational studies with mechanistic support; Level 4, clinical cohort studies with interventional or translational validation. The resulting synthesis included eight studies and was conducted as a structured qualitative comparative framework rather than a quantitative meta-analysis. A formal quality appraisal of the included studies was performed using the Joanna Briggs Institute checklist for human cohort studies and SYRCLE’s tool for preclinical studies, with the appraisal reported in **Supplementary Table 2** and the study-selection flow diagram shown in **Supplementary Figure 2**. Because this was a single-author study, study selection and appraisal were conducted by one reviewer rather than independently by multiple reviewers.

## 3 Results

To address the study’s aims, research hotspots were mapped using keyword co-occurrence analysis and thematic mapping, intellectual foundations were examined through reference co-citation analysis, clinical and translational trends were assessed using overlay visualization, thematic evolution, and annual scientific production trends, and biological interpretation was developed through cross-cohort functional integration of representative microbiome–ICI studies.

### 3.1 Annual scientific production and clinical translation

**Figure 1:**
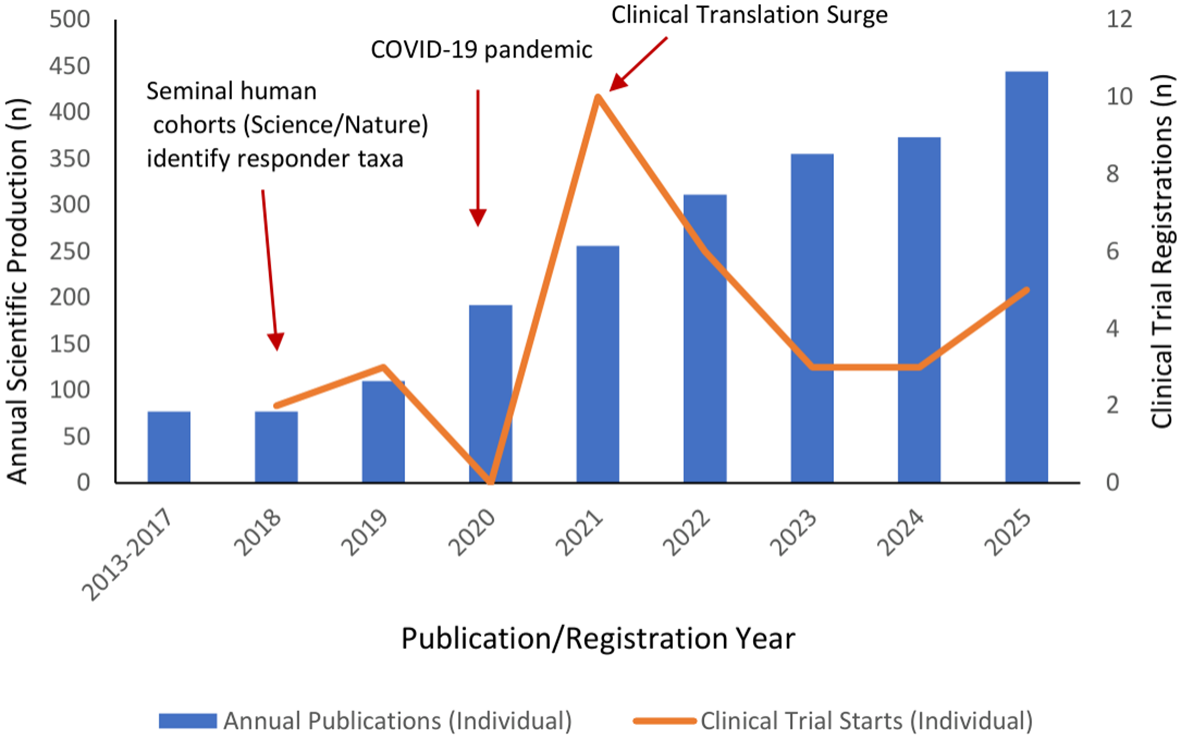
Annual scientific production and clinical translational momentum (2013–2025). Bars represent the annual number of publications indexed in the Web of Science Core Collection (left y-axis). The orange line indicates the annual initiation of interventional clinical trials registered on ClinicalTrials.gov (right y-axis). Landmark human cohort studies in 2018 catalyzed a significant translational surge, peaking in 2021 with ten new clinical trial registrations.

To evaluate the field’s clinical maturity, a dual-axis mapping approach was employed to assess scientific publication volume and interventional clinical trial momentum (**Figure 1**). The annual publication output followed a steady exponential growth trajectory between 2013 and 2025, reaching a peak of 444 publications in 2025. The initial period, from 2013 to 2017, was characterized by relatively low scientific output, reflecting the foundational preclinical phase of microbiome–immunotherapy research.

However, the secondary axis reveals a distinct translational narrative. Following the publication of seminal human cohort studies in 2018 that established an initial correlation between specific microbial taxa and ICI efficacy, clinical interest was rapidly catalyzed. A secondary search of ClinicalTrials.gov identified 31 interventional trials that specifically targeted the gut microbiome to enhance ICI outcomes. Notably, the field experienced a significant translational surge in 2021, when clinical trial initiations reached their peak (n = 10). This distinct lag between the 2018 scientific discoveries and the 2021 interventional peak illustrates the critical window required for preclinical hypotheses to mature into clinical testing. Collectively, this upward trajectory supports that research on the gut microbiome–ICI axis has successfully transitioned from descriptive taxonomy and observational correlations to an active, therapeutically driven phase of cancer research.

Among the 31 identified interventional trials (**Supplementary Table 1**), fecal microbiota transplantation (FMT) represented the dominant translational strategy (n = 24, 77%), particularly in melanoma, non-small cell lung cancer, and gastrointestinal malignancies. Most studies were early-phase trials focused on overcoming primary or acquired resistance to immune checkpoint inhibitors, enhancing therapeutic efficacy, or mitigating immune-related toxicity, the latter represented by three FMT trials specifically targeting ICI-induced colitis. Probiotic and live biotherapeutic approaches (n = 7), including *Akkermansia muciniphila*–based products and defined microbial consortia (MaaT013, MET-4), reflected a gradual transition from observational microbiome association studies toward therapeutic microbiome engineering. Notably, no interventional studies based on dietary modulation, metabolite-based formulations, or antibiotic stewardship were identified, indicating that the pipeline remains anchored to ecosystem-transfer and live-organism strategies.

### 3.2 Research hotspots: keyword co-occurrence network

**Figure 2:**
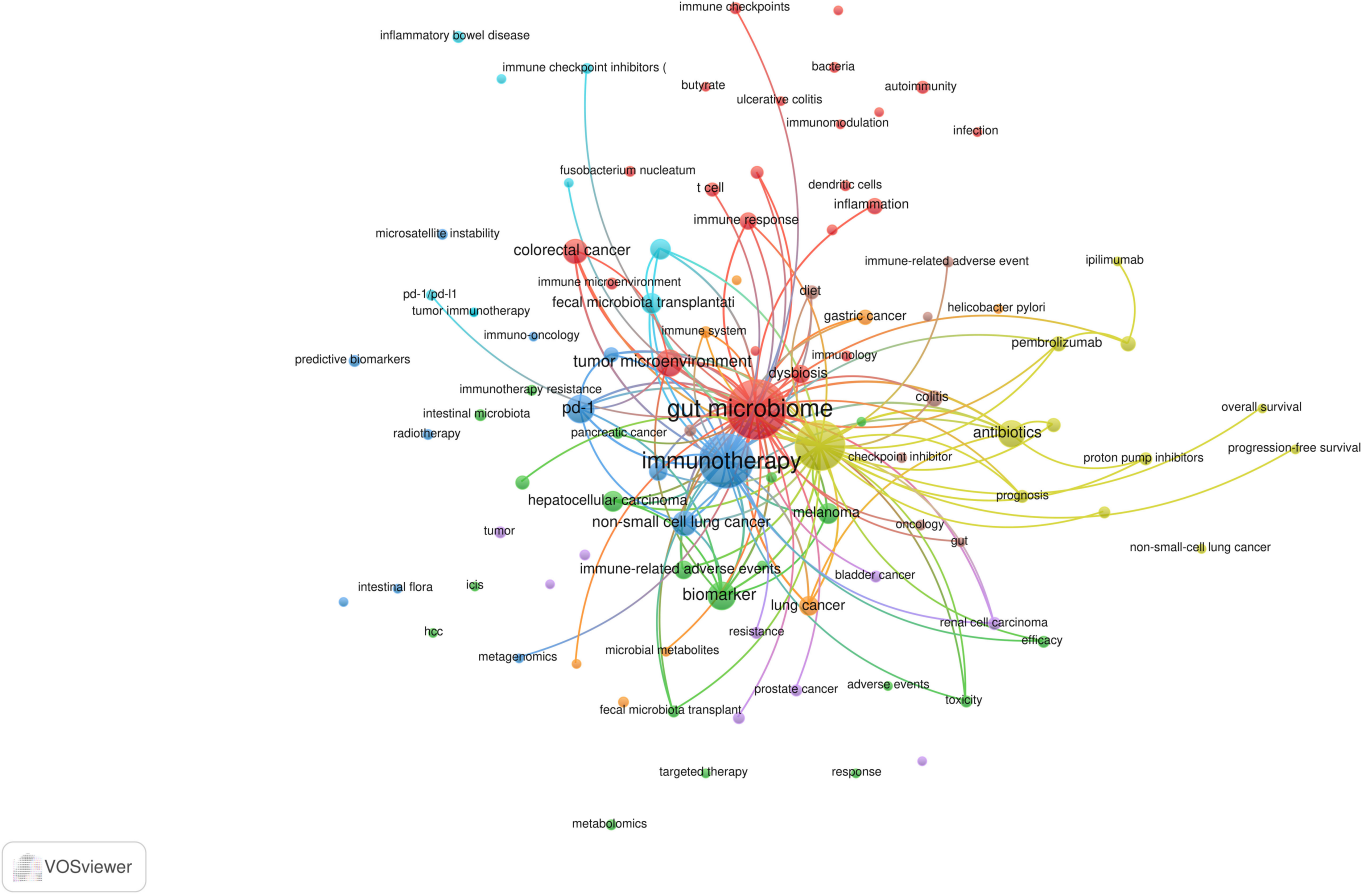
Keyword co-occurrence network of gut microbiome–immune checkpoint inhibitor research (2013–2025). Network visualization of author keyword co-occurrences was generated using bibliometric visualization tools based on 2,195 publications retrieved from the Web of Science Core Collection. A minimum occurrence threshold of ≥10 was applied, yielding 100 high-frequency keywords organized into eight clusters and connected by 1,132 inter-keyword links. Node size represents the frequency of keyword occurrence, link thickness reflects the co-occurrence strength, and colors indicate cluster membership. The network illustrates the conceptual structure and thematic interconnections within gut microbiome–immune checkpoint inhibitor research.

Keyword co-occurrence analysis of author keywords identified 100 high-frequency terms (minimum occurrence ≥10) organized into eight clusters with 1,132 inter-keyword links, indicating a densely connected conceptual structure (**Figure 2**). The network was centered on core terms related to immunotherapy and the gut microbiome, which acted as major hubs linking multiple thematic areas. The surrounding clusters captured checkpoint-related terminology (e.g., PD-1/PD-L1/CTLA-4, and ICI concepts), oncology contexts across major tumor types (e.g., NSCLC, melanoma, colorectal cancer, and hepatocellular carcinoma), and microbiome modulation themes, including antibiotics and intervention-oriented concepts such as fecal microbiota transplantation and probiotics. Additional clusters reflected clinically oriented endpoints and mechanisms, including biomarkers, prognosis and survival, immune responses, inflammation, and immune-related adverse events. Overall, the high link density and multi-cluster structure suggest that microbiome–ICI research is anchored in immunotherapy mechanisms while expanding into cancer-specific applications and intervention-driven, clinically relevant questions.

### 3.3 Temporal trends and emerging themes: overlay visualization by average publication year

**Figure 3:**
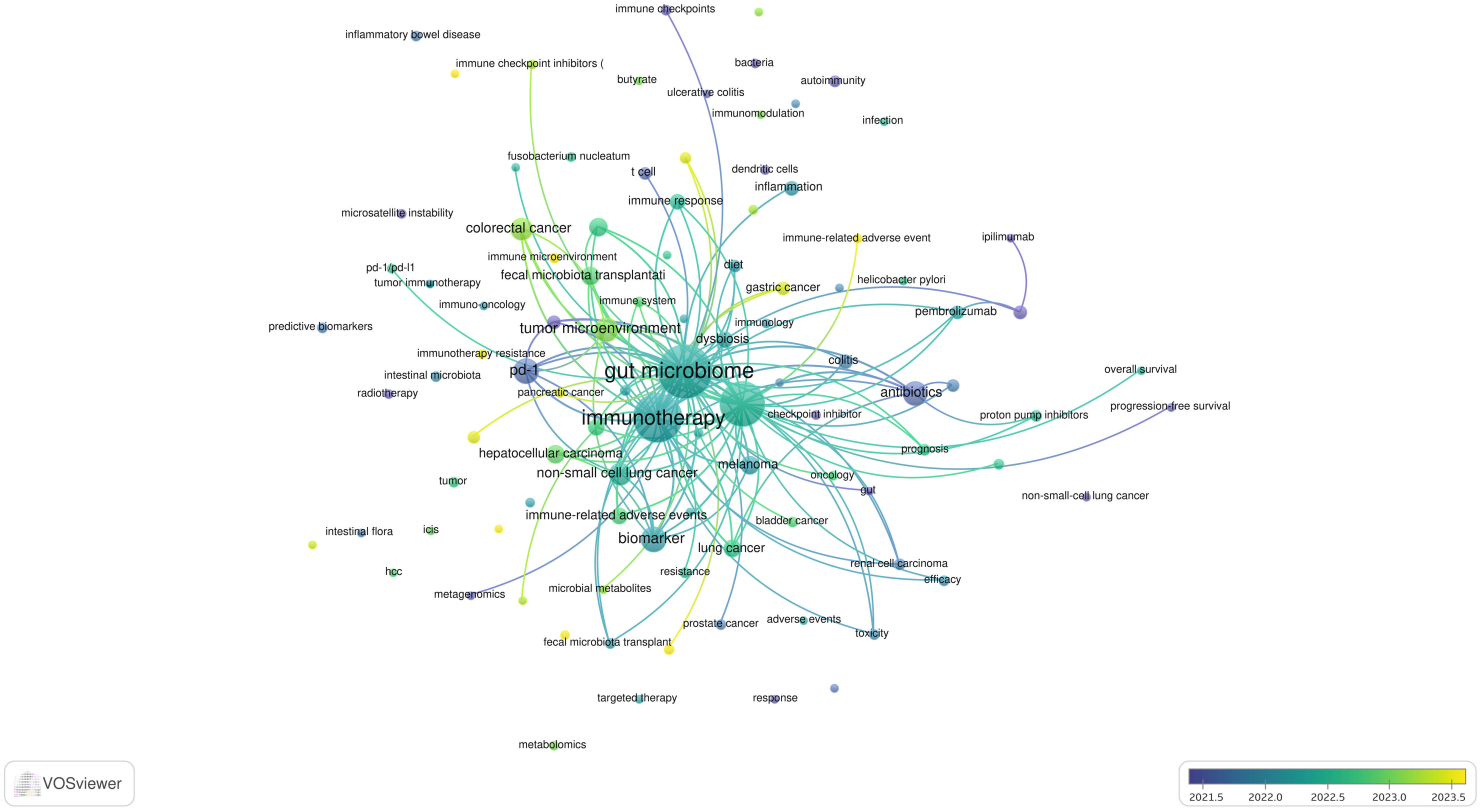
Overlay visualization of keyword co-occurrence by average publication year (2021–2025). Overlay visualization of author keyword co-occurrence was generated using bibliometric visualization tools, in which node colors represent the average publication year of each keyword. The color gradient ranges from blue/purple (earlier average publication years) to green and yellow (more recent average publication years), as indicated by the legend (approximately 2021.5–2023.5). Node size reflects keyword frequency, and link thickness represents co-occurrence strength. Earlier-emerging terms are predominantly associated with specific immune checkpoint inhibitor (ICI) agents (e.g., ipilimumab, pembrolizumab), treatment modifiers (e.g., antibiotics, proton pump inhibitors), and outcome-oriented concepts (e.g., overall survival, progression-free survival). More recent terms cluster around microbiome modulation strategies (e.g., fecal microbiota transplantation, microbial metabolites) and advanced profiling techniques (e.g., metagenomics, metabolomics), illustrating the temporal evolution of research priorities within the gut microbiome–immune checkpoint inhibitor field from foundational outcome studies toward targeted interventions.

An overlay visualization by average publication year across the 2021–2025 study period (**Figure 3**) highlighted a clear temporal gradient across the keyword network, separating earlier mechanistic and drug-focused concepts from newer translational themes. Older terms within this timeframe (cool colors, ~2021.5) were concentrated around specific checkpoint inhibitors (e.g., ipilimumab and pembrolizumab), treatment modifiers (e.g., antibiotics and proton pump inhibitors), and outcome-oriented endpoints (e.g., overall survival and progression-free survival). In contrast, more recent terms (warm colors, ~2023.5) clustered around microbiome modulation and advanced analytical techniques. These clinically actionable and emerging directions include fecal microbiota transplantation, microbial metabolites (e.g., butyrate), and metagenomics and metabolomics. Collectively, the overlay indicates a rapid evolution in the field, moving from foundational outcomes and drug-interaction studies to targeted interventions and multi-omics microbiome research. This transition in published research vocabulary aligns chronologically with the current secondary clinical analysis; interventional trial registrations peaked early in the study period (2021), driving the subsequent migration of themes, such as fecal microbiota transplantation, into the current research foreground (**Figure 1**).

### 3.4 Mapping the intellectual transition: Sankey diagram analysis of research hotspots (2013–2025)

**Figure 4:**
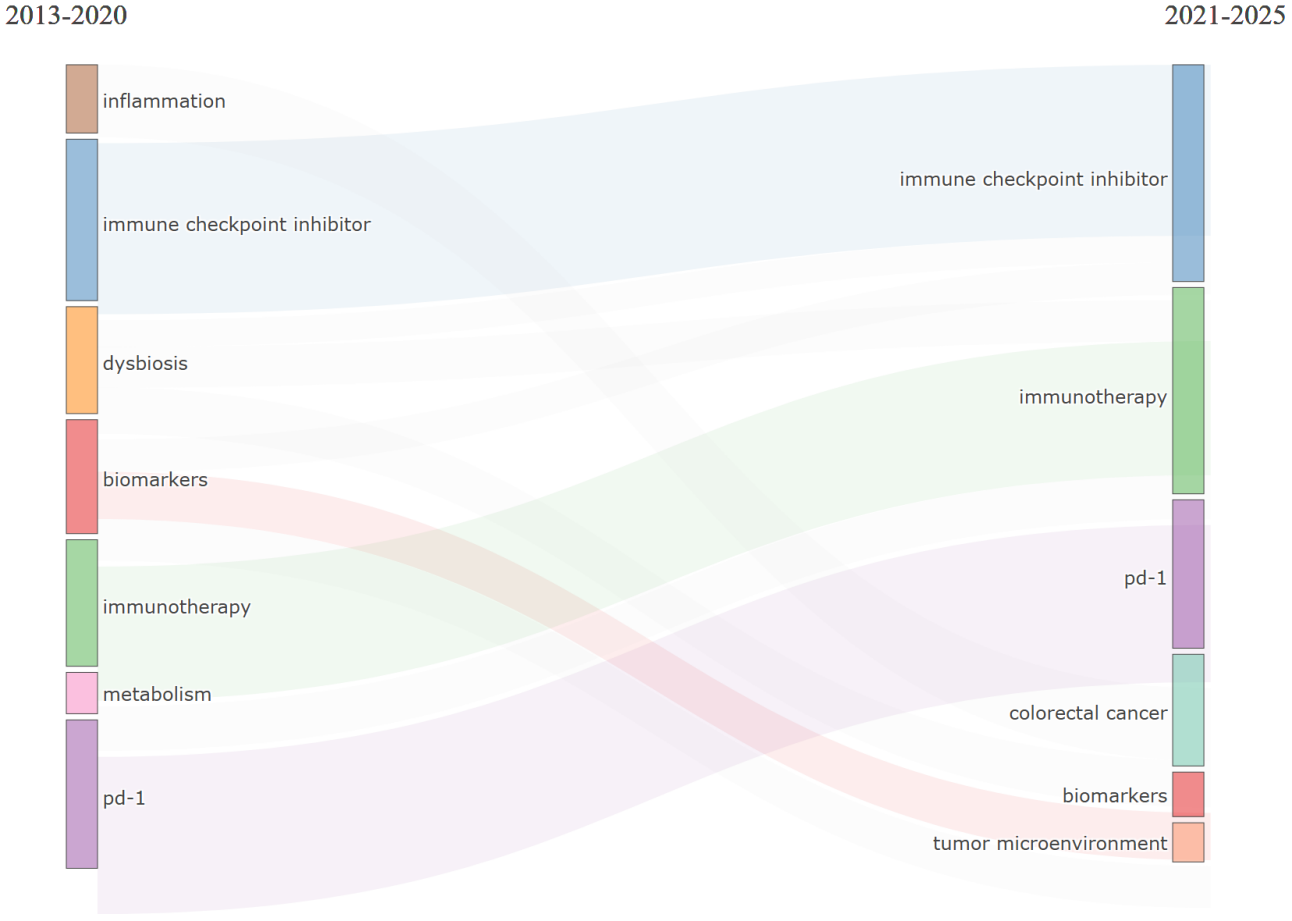
Thematic evolution of research hotspots in gut microbiome–immune checkpoint inhibitor studies (2013–2025). Sankey diagram illustrating the longitudinal evolution of major research themes across two temporal phases: 2013–2020 (left) and 2021–2025 (right). Themes were identified using thematic evolution analysis in the bibliometrix R package (Biblioshiny) based on author keywords. Rectangles represent the dominant themes within each period, and flow widths indicate the strength of thematic continuity and conceptual linkages between phases. The diagram suggests the progression of early foundational themes, such as inflammation, dysbiosis, metabolism, and PD-1, toward later emphasis on immune checkpoint inhibitors, immunotherapy, colorectal cancer, biomarkers, and the tumor microenvironment, reflecting the field’s transition from mechanistic exploration to clinically oriented and tumor-specific applications.

The longitudinal transformation of the gut microbiome–ICI research landscape was visualized using thematic evolution analysis, mapping the progression and convergence of key research nodes across two distinct temporal windows: Phase I (2013–2020) and Phase II (2021–2025; **Figure 4**). During the initial period, the intellectual structure was heavily anchored in characterizing fundamental biological mechanisms, with “inflammation,” “dysbiosis,” and “metabolism” serving as significant early hotspots. These themes reflect foundational efforts to define the host–microbiome interface and its systemic influence on the efficacy of ICIs. Notably, “biomarkers” and “PD-1” acted as central pillars during this phase, highlighting the early-stage priority of identifying clinical indicators for patient stratification.

The transition to the contemporary window (2021–2025) revealed a distinct shift from generalized mechanistic exploration toward applied clinical translation and tumor-specific contexts (**Figure 4**). Early interest in “inflammation” and “metabolism” largely converged into investigations of the “tumor microenvironment,” indicating maturation of the field toward localized immune-microbiome interactions within the oncology setting. Furthermore, the evolution of the “dysbiosis” theme into broader “immunotherapy” and “immune checkpoint inhibitor” clusters suggests that the clinical focus has shifted toward therapeutic modulation of these microbial imbalances to enhance treatment outcomes. The second phase also highlights the rise of “colorectal cancer” as a prominent clinical indication, reflecting the expansion of microbiome-related ICI research beyond foundational tumor types, such as melanoma. This thematic flow underscores a field that has successfully transitioned from establishing simple biological correlations to actively investigating the complex interplay within the “tumor microenvironment,” with “biomarkers” remaining a persistent bridge connecting both eras of research.

### 3.5 Strategic thematic mapping: assessing the maturity and evolution of research hotspots

**Figure 5:**
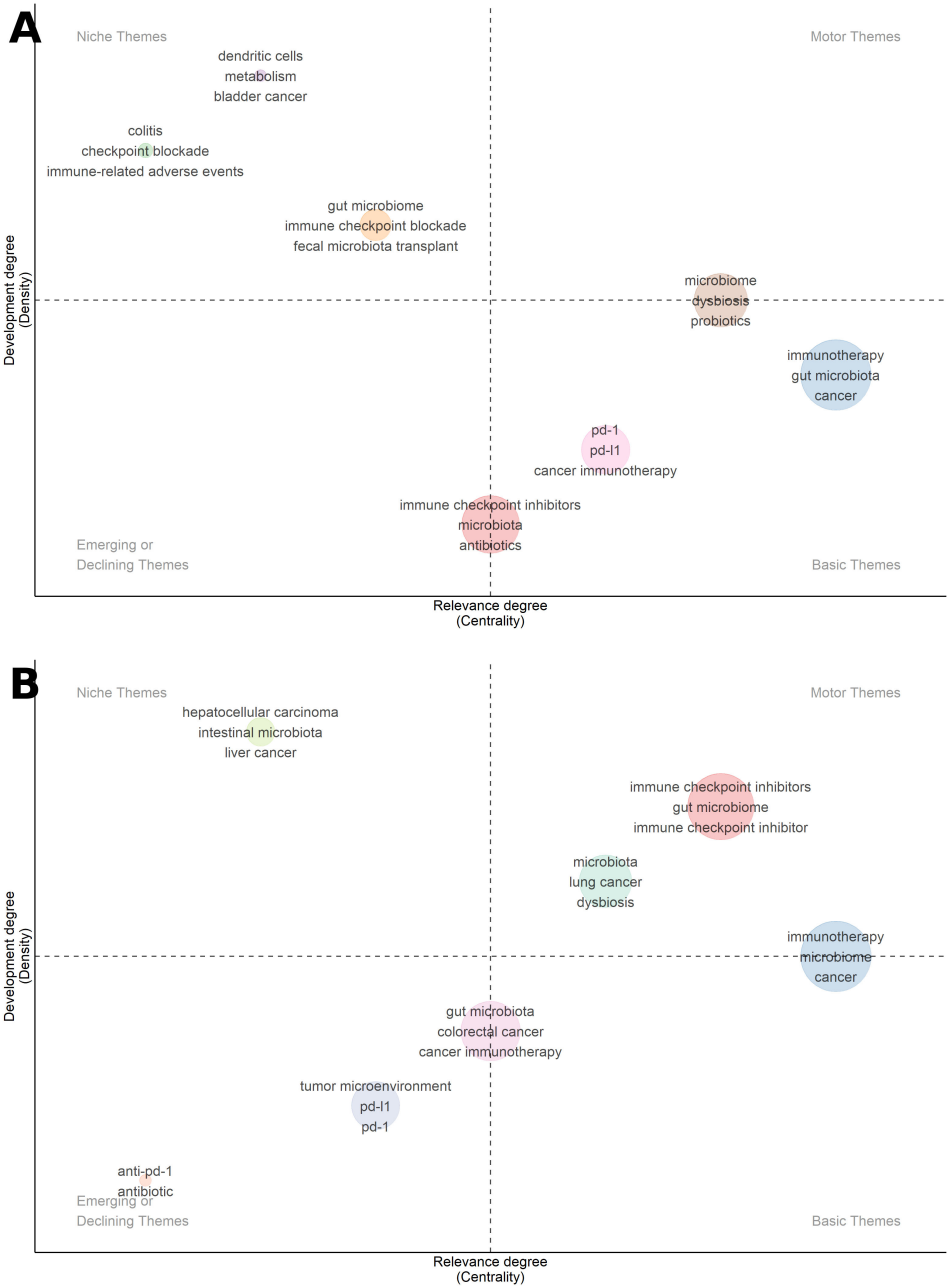
Strategic thematic maps of gut microbiome–immune checkpoint inhibitor research. **(A)** Phase I (2013–2019) and **(B)** Phase II (2020–2025). Maps were generated using the bibliometrix R package (Biblioshiny), plotting thematic clusters by centrality (x-axis; relevance) and density (y-axis; development). The four quadrants represent motor (upper right), niche (upper left), emerging/declining (lower left), and basic/transversal themes (lower right). Bubble size reflects theme frequency. Between the two phases, the field suggests clear thematic consolidation and clinical specialization, with concepts such as immune checkpoint inhibitors and gut microbiome shifting from foundational/transitional roles in Phase I to highly developed motor themes in Phase II.

Strategic thematic mapping revealed a significant maturation of the research field, evolving from foundational mechanistic studies in Phase I (2013–2019) to specialized clinical applications in Phase II (2020–2025; **Figure 5**). In the initial phase, research was characterized by a focus on the fundamental intersection of the “microbiome” and “cancer,” which emerged as primary motor themes driving the intellectual structure. During this period, the framework was anchored by basic, transversal themes such as “immunotherapy” and “gut microbiota,” while more specialized niche investigations focused on “dendritic cells” and “metabolism” (**Figure 5A**). Notably, “fecal microbiota transplant” and “immune checkpoint blockade” were situated near the central axis as emerging themes, reflecting the early-stage clinical interest and the initial controversies surrounding microbial modulation highlighted in the foundational literature.

In Phase II, the thematic landscape demonstrated a clear trajectory toward clinical translation and consolidation of the microbiome as a central pillar of immunotherapy (**Figure 5B**). The cluster representing “gut microbiome” and “immune checkpoint inhibitors” migrated firmly into the motor theme quadrant, exhibiting high centrality and density, which indicate its role as the dominant engine of current research. This maturation is further evidenced by the emergence of specialized niche themes, such as “hepatocellular carcinoma” and “liver cancer,” suggesting a shift from broad proof-of-concept studies to organ-specific clinical indications. Furthermore, the transition of foundational terms such as “anti-PD-1” toward the emerging or declining quadrant suggests that research has moved beyond basic monotherapy pathways to prioritize complex microbial interactions and “dysbiosis” as predictive and therapeutic hotspots. This evolution underscores the transition from establishing a biological link to optimizing clinical outcomes and managing treatment-related complexities in contemporary research landscapes. The movement of fecal microbiota transplantation into the motor theme quadrant served as a bibliometric anchor for the 31 interventional trials identified in this study, supporting the field’s shift into a dominant translational engine.

### 3.6 Co-citation network

**Figure 6:**
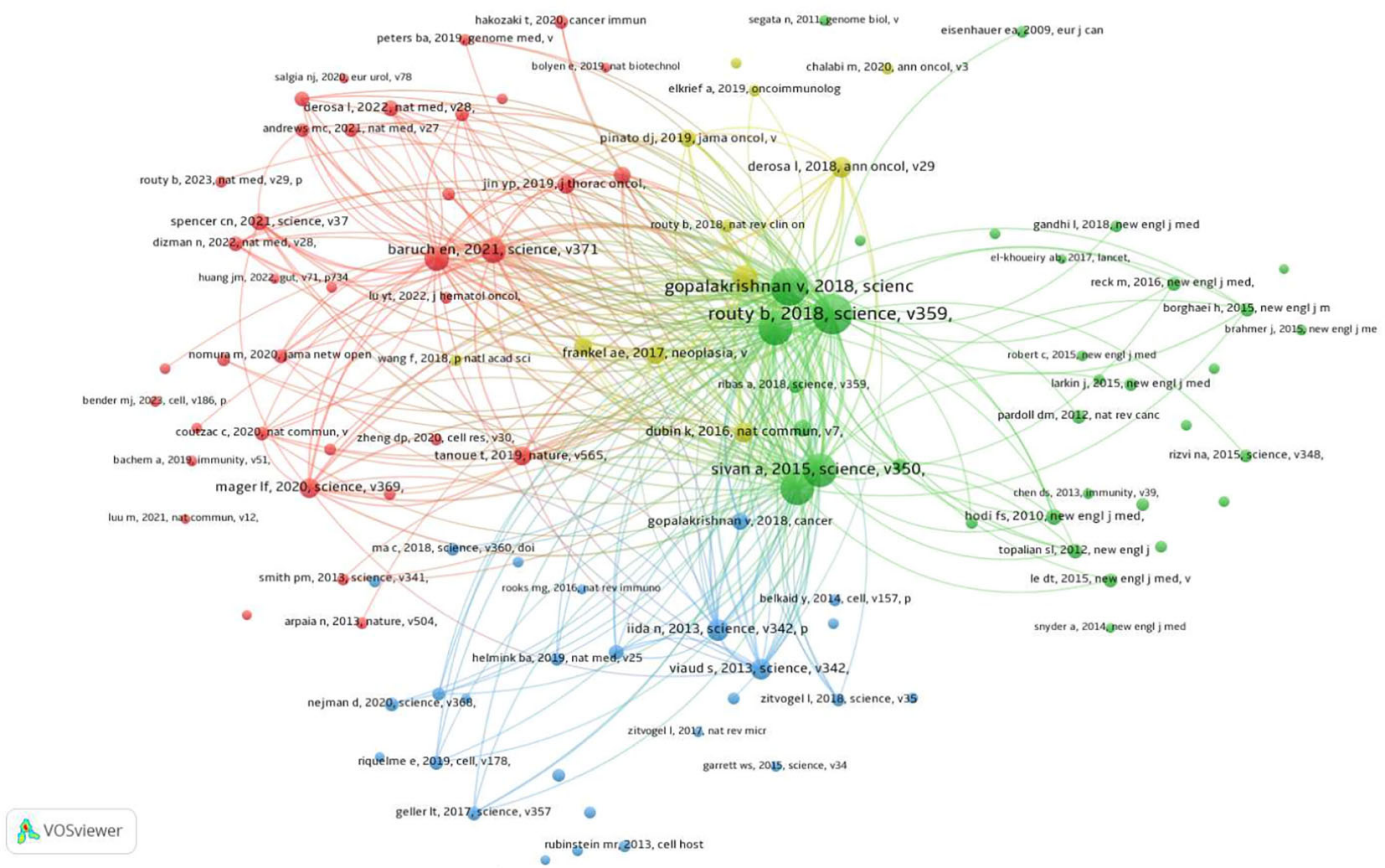
Network visualization of document co-citation analysis. The co-citation network of 110 highly cited references was visualized using bibliometric visualization tools. Node size reflects the citation frequency of each document, and the thickness of the connecting lines indicates the strength of the co-citation relationship. The network is divided into four closely interlinked thematic clusters: a central hub of foundational microbiome–immune checkpoint inhibitor (ICI) studies published between 2015 and 2018 (green); established clinical frameworks for checkpoint immunotherapy (teal); recent translational and clinical cohort studies (red); and early preclinical mechanistic research (dark blue). Seminal papers, such as those by Routy et al., Gopalakrishnan et al., and Sivan et al., anchor the central network, illustrating a highly cohesive and interdependent knowledge base driving the field.

As illustrated in **Figure 6**, the reference co-citation network comprises 110 highly cited references (minimum citations = 70) connected by 5,927 co-citation links with a total link strength of 135,262, revealing four major thematic clusters. The central cluster (green, **Figure 6**) features the largest nodes in the network, representing the most influential and highly connected hub papers published between 2015 and 2018. These include Sivan et al. (12) and landmark microbiome–ICI studies such as those of Routy et al. (11) and Gopalakrishnan et al. (9), highlighting their foundational role in establishing the link between the gut microbiome and ICI efficacy. The second cluster (teal) captures the clinical foundation of checkpoint immunotherapy, including seminal PD-1/PD-L1 and CTLA-4 clinical and mechanistic studies, indicating that microbiome–ICI research is firmly grounded in established immunotherapy frameworks. The third cluster (red) was relatively enriched with more recent translational and clinically oriented studies (predominantly 2019–2022), consistent with the expansion of microbiome modulation strategies and clinical evaluations. The fourth cluster (dark blue) comprises earlier mechanistic and preclinical microbiome–immunity studies, reflecting experimental work that informed biological hypotheses later tested in clinical settings. Notably, the dense intercluster linkages throughout the network (**Figure 6**) suggest that the field evolved around a coherent and interdependent knowledge base rather than a disconnected subfield.

### 3.7 Multi-layer functional integration of microbiome–ICI interactions

To provide biological reinterpretation for the bibliometric findings, a multi-layer functional integration was performed by synthesizing taxonomic, pathway-level, and metabolic evidence from representative clinical and preclinical studies. Qualitative cross-cohort comparison of landmark clinical datasets (9–11, 26, 27) suggested recurrent enrichment of multiple immunologically relevant taxa in ICI responders, including *Akkermansia muciniphila*, members of the Ruminococcaceae family, *Enterococcus* spp., *Alistipes* spp., and *Collinsella aerofaciens*, whereas non-responder-associated taxa exhibited greater inter-cohort variability (**Supplementary Figure 1**). At the functional level, responder-associated microbiomes were characterized by metabolically supportive and immunologically favorable profiles, including short-chain fatty acid production, dendritic cell activation, and CD8^+^ T-cell priming, while non-responders showed features consistent with dysbiosis and immunosuppressive tumor microenvironments. These functional relationships are summarized in **Table 1**, linking representative microbial taxa to immune pathways and clinical outcomes across multiple levels of evidence. Importantly, these observations suggest that variability at the taxonomic level does not preclude consistency at the functional level, supporting a model of functional convergence in microbiome–ICI interactions in which distinct microbial communities engage overlapping immunological pathways relevant to ICI response.

**Table 1:** Functional pathways linking microbiome composition to immune checkpoint inhibitor response and resistance.

Functional pathway (microbe → mechanism → outcome)	Representative study	Evidence level*	Rationale
*Akkermansia muciniphila* → immune activation → ICI response	Routy et al., 2018 (Science)	4	Clinical cohorts and fecal microbiota transplantation (FMT) experiments demonstrated restoration of anti–PD-1 efficacy.
SCFA-producing taxa → CD8^+^ T-cell activation → ICI response	Gopalakrishnan et al., 2018 (Science)	3	Mechanistically supported human observational cohorts linked SCFA-associated Ruminococcaceae to enhanced CD8^+^ T-cell tumor infiltration.
*Bifidobacterium* spp. → dendritic cell maturation → ICI response	Sivan et al., 2015 (Science)	2	Preclinical *in vivo* models demonstrated enhanced dendritic cell priming and tumor-specific T-cell activation following microbial administration.
*Bacteroides fragilis* → Th1 immune priming → ICI response	Vétizou et al., 2015 (Science)	2	Murine models showed microbiome-dependent Th1 responses and dendritic cell maturation required for CTLA-4 blockade efficacy.
Inosine-producing commensal taxa → T-cell activation → ICI response	Mager et al., 2020 (Science)	2	Preclinical studies demonstrated that microbiome-derived inosine enhances systemic T-cell activation and improves ICI efficacy.
Defined commensal consortia → immune homeostasis → ICI response	Tanoue et al., 2019 (Nature)	2	Preclinical *in vivo* models showed that defined microbial consortia promote CD8^+^ T-cell-mediated immune homeostasis and antitumor responses.
Antibiotic-induced dysbiosis → immune suppression → ICI resistance	Routy et al., 2018 (Science)	4	Clinical evidence demonstrated that antibiotic exposure correlates with reduced progression-free and overall survival across multiple cohorts.
Dysbiotic pathobionts → T-cell exhaustion → ICI resistance	Matson et al., 2018 (Science)	3	Human cohort analyses revealed associations between dysbiosis, impaired memory T-cell responses, and primary resistance to ICI therapy.

*Evidence levels are defined in the Methods section (Section 2.5).

### 3.8 Translational framework of microbiome-mediated ICI response and resistance

**Figure 7:**
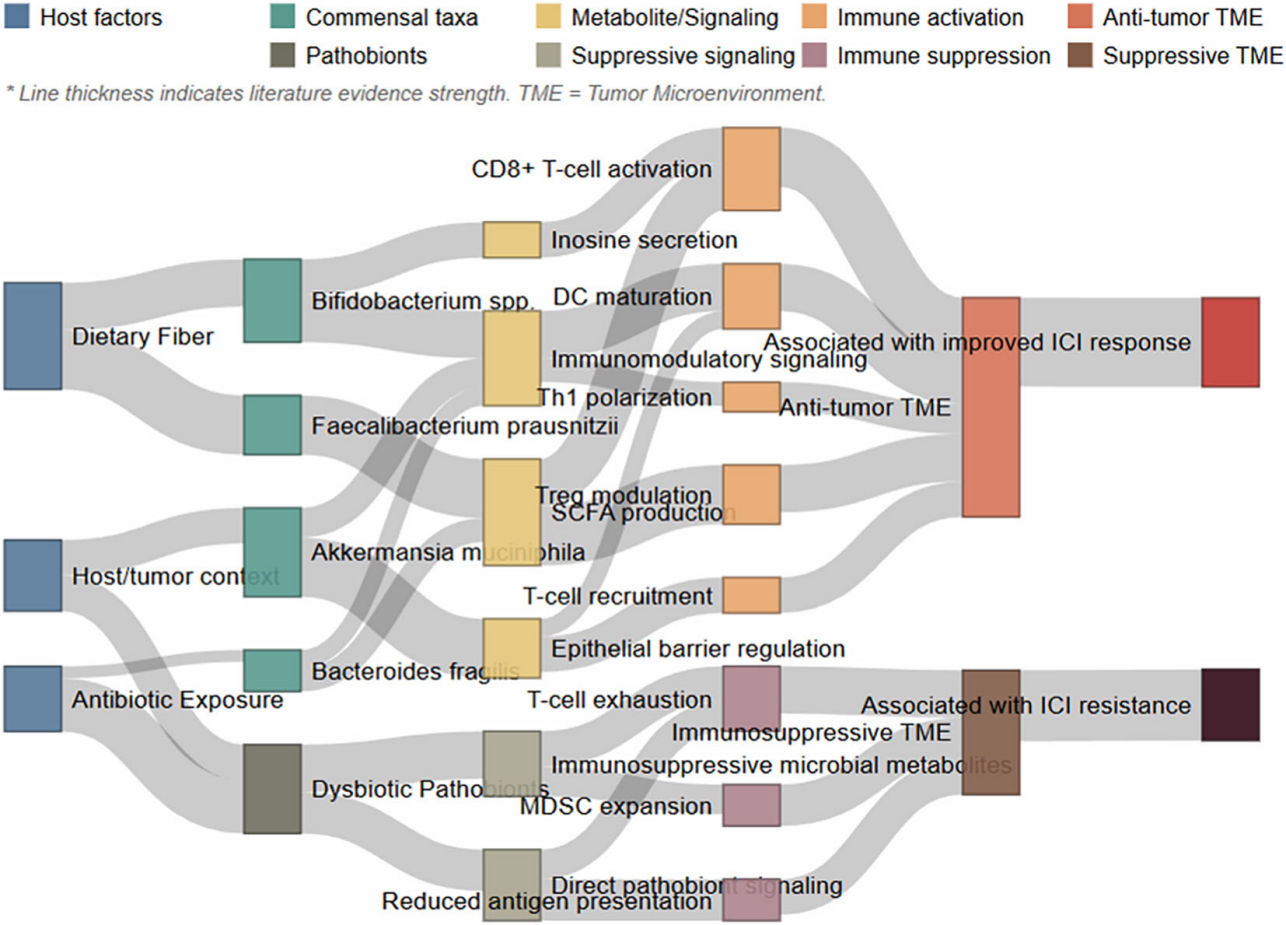
Conceptual framework of microbiome-mediated immune checkpoint inhibitor (ICI) outcomes. Sankey diagram illustrating a literature-informed framework integrating bibliometric and functional evidence to represent relationships between host factors, microbial composition, metabolite signaling, immune modulation, and clinical response. Response-associated pathways include commensal taxa and microbial metabolites (e.g., short-chain fatty acids, inosine) linked to immune activation (e.g., CD8^+^ T-cell responses), whereas resistance-associated pathways reflect dysbiosis, antibiotic exposure, and immunosuppressive mechanisms (e.g., T-cell exhaustion). The relative thickness of connections represents the strength of supporting evidence across the literature.

Building on the integrated functional findings, a literature-informed conceptual framework was developed to illustrate the mechanistic pathways linking host factors, microbial composition, metabolite signaling, and immune responses to ICI outcomes (**Figure 7**). The framework integrates the bibliometric structure with representative biological evidence, providing a unified interpretation of microbiome–ICI interactions.

In the response-associated arm, host-related factors such as dietary fiber and tumor context are linked to the enrichment of commensal taxa, including *Bifidobacterium* spp., *Faecalibacterium prausnitzii*, and *Akkermansia muciniphila*. These taxa converge on metabolite-driven pathways, including short-chain fatty acid production and inosine secretion, which promote immune activation through dendritic cell maturation, Th1 polarization, and CD8^+^ T-cell activation, ultimately supporting an immunostimulatory tumor microenvironment and improved ICI response. In contrast, antibiotic exposure and dysbiosis are associated with pathobiont expansion, leading to immunosuppressive microbial metabolites, impaired antigen presentation, T-cell exhaustion, and myeloid-derived suppressor cell expansion. These factors collectively drive an immunosuppressive tumor microenvironment and ICI resistance. The relative thickness of the connections in the framework reflects the strength of supporting evidence across the literature, highlighting that microbiome–ICI interactions are driven by functionally convergent pathways rather than individual taxa alone.

## 4 Discussion

The central message of this critical synthesis is not simply that gut microbiome–ICI research has grown, but that it has entered a contested translational phase in which apparent inconsistencies at the taxonomic level may reflect convergence at the functional level. The field has clearly moved beyond early association studies, as reflected by the increase in interventional trial activity after landmark human cohorts were published in 2018 and the peak in trial initiation in 2021 (**Figure 1**) (9–11). However, this rapid progress has not been matched by consensus on what should be measured, how it should be measured, or which microbiome signals are clinically actionable (13, 31–34). In this sense, the defining feature of the 2013–2025 period is twofold: rapid clinical ambitions alongside unresolved methodological and biological inconsistencies. The key contribution of this study is the integration of bibliometric evidence with cross-cohort functional interpretation, showing that persistent taxonomic variability across microbiome–ICI studies is consistent with functional convergence rather than biological contradiction, with direct implications for biomarker development and clinical trial design.

### 4.1 From taxonomy-driven discovery to mechanism- and intervention-oriented research

A major contribution of the current mapping analyses is the demonstration that the field’s priorities have shifted from “who is there?” to “what are they doing, and can we manipulate it therapeutically?” Early research was dominated by checkpoint terminology, canonical tumor types (especially melanoma and NSCLC), and descriptive microbiome-response associations, whereas more recent themes cluster around FMT, probiotics and prebiotics, microbial metabolites (e.g., butyrate), biomarkers, survival endpoints, and treatment modifiers such as antibiotics and proton pump inhibitors (**Figures 2**-**5**) (9–21). This transition is not merely semantic; it reflects the maturation of scientific questions.

For a clinical and immunology audience, this thematic shift carries clear biological and interpretive significance, suggesting that the field’s apparent inconsistencies may be explained by shared functional outputs rather than divergent microbial biology. The early focus on taxa-level associations (including recurrent attention to *A. muciniphila* and *Bifidobacterium*) has helped establish the plausibility that gut microbes can influence antitumor immunity (11, 12). The newer emphasis on metabolites and tumor microenvironment-related themes suggests increasing recognition that microbial effects are likely mediated through immune-modulatory functions—for example, altered antigen presentation, T-cell priming, dendritic cell activation, cytokine signaling, epithelial barrier integrity, and metabolite-driven immune tone—rather than through the simple presence or absence of single taxa (14–19, 33, 35). This interpretation is further supported by cross-cohort comparison of landmark clinical studies (**Supplementary Figure 1**), which suggests that although specific taxa differ between cohorts, responder-associated microbiomes consistently feature immunomodulatory commensals, reinforcing the robustness of these functional patterns. In practical terms, this shifts the translational focus from a taxonomic “signature hunt” toward functionally informed intervention strategies, including metabolite modulation, defined microbial consortia, and context-specific microbiome remodeling (13–21, 33, 35).

This interpretation also explains why biomarkers remain a persistent bridge theme across phases in thematic evolution analyses (**Figure 4**). The field has generated substantial evidence that microbiome composition and ecology matter. However, the clinically relevant challenge is patient stratification, i.e., identification of which microbial states, functions, or host–microbiome interactions predict benefit, resistance, or toxicity under specific ICI regimens and tumor contexts (13, 31–34, 36). Importantly, the present study extends this thematic transition beyond bibliometric mapping by integrating representative taxonomic, functional, and metabolic evidence into a unified interpretive framework. The multi-layer functional integration suggests that microbiome–ICI interactions converge on a limited set of immunologically relevant pathways, including short-chain fatty acid production, dendritic cell activation, T-cell priming, and tumor microenvironment modulation. This perspective suggests that apparent inconsistencies at the taxa level may reflect functional redundancy rather than true biological contradiction. In this context, the proposed framework provides a mechanistic bridge linking microbial composition to immune outcomes and supports a shift from taxonomy-driven associations toward function-based and pathway-oriented strategies for biomarker development and therapeutic intervention.

### 4.2 A central controversy: the crisis of consistency

A central controversy highlighted by the current mapping is the crisis of consistency at the taxonomic level. Across the literature, many studies report “response-associated” bacteria; however, reproducibility across cohorts remains limited (9–11, 13, 31, 32, 34, 36). This tension persists despite a strongly interconnected keyword network (eight clusters linked by 1,132 inter-keyword links; **Figure 2**) and accelerating interventional activity (**Figure 1**), with biomarker-, dysbiosis-, FMT-, and outcome-centered themes continuing to evolve in parallel (**Figures 2**-**5**). These trends indicate ongoing uncertainty rather than convergence within a settled clinical framework.

Several factors likely contribute to this inconsistency. Biological heterogeneity is substantial: tumor type, disease stage, geography, diet, comorbidities, and prior treatments all shape microbiome structure and host immunity (13, 31, 32, 34). Methodological heterogeneity is equally important, including 16S rRNA sequencing versus shotgun metagenomics, differences in taxonomic classifiers and reference databases, variable sample collection timing, inconsistent response definitions, and incomplete control of major confounders, especially antibiotics, proton pump inhibitors, corticosteroids, and nutritional status (28, 31, 32, 37, 38). Under these conditions, discordant taxonomic findings are expected.

Critically, the cross-cohort functional integration performed in this study (Section 3.7; **Table 1**; **Supplementary Figure 1**) provides a complementary biological interpretation. While the specific taxa enriched in responders differ across cohorts, the immunological pathways implicated converge consistently around short-chain fatty acid production, dendritic cell maturation, and CD8^+^ T-cell priming. This pattern suggests that taxonomic discrepancies across studies do not necessarily reflect conflicting biology, but rather alternative microbial routes to similar immunological outcomes. A key implication is that the field may overinterpret taxonomic disagreements as biological contradictions. Distinct microbial communities can exhibit overlapping metabolic and immune functions. Future convergence may therefore emerge less from repeating species-level association studies and more from linking microbial ecology to shared functions (e.g., short-chain fatty acid production, bile acid metabolism, inflammatory signaling modulation) and to measurable immune phenotypes relevant to ICI response (14–19, 28, 33, 35, 36). This functional reframing offers a coherent explanation for the observed “crisis of consistency” and supports a shift toward function-based interpretation of microbiome–ICI interactions.

Although the functional convergence framework provides a biologically plausible interpretation of taxonomic variability across microbiome–ICI studies, this conclusion should be considered hypothesis-generating rather than definitive. Alternative explanations for inconsistent microbial signatures remain possible, including the sources of biological and methodological heterogeneity noted above, as well as differences in bioinformatic pipelines and statistical approaches. In addition, similarities in inferred functional pathways may partly reflect limitations of pathway annotation methods or preferential reporting of immunologically relevant mechanisms. Therefore, functional convergence should be viewed as one explanatory model that requires further validation through standardized prospective cohorts, integrated multi-omics analyses, and mechanistically controlled experimental studies.

### 4.3 Causality remains the translational bottleneck

Another major controversy is the gap between association-rich evidence and clinically useful causality. The dual-axis trend analysis and the thematic shift toward interventions indicate that the field has already moved to clinical testing (**Figures 1**, **3**-**5**) (20, 21). Observational studies can identify strong correlations, but causal inference is constrained by multiple biological and methodological challenges. Importantly, reverse causation cannot be excluded, as microbiome differences observed in responders and non-responders may represent consequences of underlying host immune status, tumor progression, treatment exposure, or disease-related changes rather than direct drivers of ICI efficacy. Additional confounders, including baseline immunity, tumor type, geographic variation, dietary patterns, antibiotic exposure, proton pump inhibitor use, corticosteroid treatment, prior anticancer therapies, and disease burden, may independently influence both microbiome composition and clinical outcomes (13, 27, 28, 31, 32, 34, 36–38). Furthermore, the methodological heterogeneity noted above — including variability in sequencing platforms, bioinformatic pipelines, and response definitions — further complicates cross-study comparisons and limits causal interpretation.

Preclinical mechanistic studies and microbiome transfer experiments have been essential in sustaining translational momentum because they provide biologically plausible pathways linking gut microbes to antitumor immunity (9–12, 14–19, 28–30). The co-citation network supports this interpretation by showing that translational work is anchored in both immuno-oncology foundations and microbiome–immunity mechanistic studies (**Figure 6**) (9–12, 20, 21). This represents a sign of intellectual maturity, but not yet causal readiness for routine clinical implementation. The next phase requires longitudinal, mechanistically annotated human studies and interventional trials designed to test not only efficacy, but also mechanism, durability, and context dependence (20, 21, 31, 32, 36).

Future progress in microbiome-guided immunotherapy will require well-powered randomized controlled trials (RCTs) to determine whether microbiome modulation directly improves ICI outcomes rather than simply correlating with therapeutic response. These clinical studies should be integrated with longitudinal multi-omics profiling and mechanistic animal models to identify the microbial metabolites, immune pathways, and host–microbe interactions responsible for treatment modulation. Combining rigorous clinical validation with mechanistic investigation will be essential for translating microbiome discoveries into reproducible precision oncology strategies.

### 4.4 Operational and regulatory barriers limit translation more than biological plausibility

The current results indicate strong translational momentum; however, the dominant barriers appear to be increasingly operational and regulatory. FMT has moved into the translational foreground (**Figures 3**-**5**), consistent with the increase in interventional trials (**Figure 1**) (20, 21). However, FMT in oncology raises well-recognized concerns, including donor screening complexity, regulatory oversight challenges (39–41), and infection transmission risk (42). These challenges are amplified in immunocompromised patients receiving anticancer therapy (41, 42).

More broadly, microbiome-directed intervention studies remain difficult to compare because of heterogeneity in donor criteria, product preparation, dosing and timing, concomitant therapies, and endpoints (20, 21, 26, 27, 31, 32, 38, 42). This creates a paradox: the field is sufficiently mature to launch trials but is not yet standardized enough to aggregate them into robust clinical guidance. If unresolved, this mismatch may slow translation despite continued publication growth.

Additional challenges arise from differences in regulatory frameworks across jurisdictions, where microbiome-based interventions may be classified differently as biological products, drugs, or tissue-based therapies. This regulatory variability complicates harmonization of manufacturing standards, safety monitoring, and clinical implementation. Because of these clinical considerations, microbiome manipulation strategies such as FMT require particularly careful evaluation of infectious risks and long-term safety. Future clinical translation will require standardized donor-selection criteria, validated screening protocols, reproducible manufacturing procedures, and harmonized outcome measures to ensure that microbiome-based therapeutics can be evaluated consistently across trials.

### 4.5 Study limitations

This study has some limitations. First, reliance on a single database and English-language publications may have introduced database and language bias, potentially omitting studies indexed elsewhere or published in non-English journals (22, 23). Inclusion of additional databases such as Scopus, PubMed, or Embase may have broadened coverage and identified additional studies, particularly those from regional or less-cited sources. Furthermore, bibliometric mapping inherently captures scholarly attention and publication trends rather than clinical efficacy, and citation lag may underrepresent the influence of the most recent (2024–2025) high-impact studies in co-citation analyses (22–25). In addition, keyword-based analyses cannot fully capture the nuances of clinical trial designs, endpoint definitions, or biological mechanisms, and remain sensitive to author terminology and synonym variations (24, 25). Finally, the cross-cohort functional integration was conducted as a qualitative comparative synthesis rather than a quantitative meta-analysis, given the substantial methodological heterogeneity across available studies. Although this approach is appropriate for interpreting functional convergence, it does not provide pooled effect estimates and should be regarded as exploratory and hypothesis-generating. Formal risk-of-bias appraisal of the eight included primary studies was performed using the Joanna Briggs Institute checklist for human cohort studies and SYRCLE’s tool for preclinical studies (**Supplementary Table 2**). However, because the cross-cohort functional integration was designed as a qualitative interpretive synthesis rather than a quantitative meta-analysis, quality assessments were used to contextualize the strength of evidence rather than to generate weighted estimates or pooled conclusions. In addition, study selection and quality appraisal were conducted by a single author, which may introduce selection or interpretation bias despite the use of predefined criteria.

### 4.6 Future trajectory and conclusion

The principal contribution of this synthesis is the reframing of microbiome–ICI inconsistency as evidence of functional convergence rather than irreproducibility or biological contradiction. The findings of this study indicate that gut microbiome–ICI research has become an intellectually mature and clinically ambitious field but remains constrained by reproducibility, causality, and standardization challenges. The next major advances are likely to originate from function-based and mechanism-linked approaches, including multi-omic biomarker frameworks, tumor microenvironment-integrated microbiome profiling, and better-controlled longitudinal intervention studies (13, 19, 28, 33, 35, 36, 40–42). Based on the trajectories identified here, future hotspots may include defined microbial consortia or engineered live biotherapeutics, metabolite-guided modulation strategies, and personalized dietary or medication stewardship approaches used alongside ICIs to improve efficacy and reduce toxicity (13, 30, 33, 40–43).

The field is no longer limited by a lack of interest or biological plausibility; it is now limited by the rigor required to convert promising microbiome signals into reproducible, safe, and clinically actionable immuno-oncology tools. The present findings further suggest that resolving current inconsistencies will depend on prioritizing functional and mechanistic reproducibility across cohorts rather than focusing solely on individual taxa.

## Data Availability

The original contributions presented in the study are included in the article/**Supplementary Material**. Further inquiries can be directed to the corresponding author.

## Supplementary material

Supplementary Figure 1 Taxonomic signatures of immune checkpoint inhibitor response across independent clinical cohorts. Differentially abundant gut microbial taxa associated with response or non-response to immune checkpoint inhibitors (ICI) are shown across landmark clinical studies. Values represent −log_10_-transformed false discovery rate (FDR), with directional encoding such that taxa enriched in responders are plotted as positive values and those enriched in non-responders as negative values. Dotted vertical lines indicate the statistical significance threshold (FDR < 0.05). Taxa identified across multiple independent cohorts are indicated.
