# Supplementary figures and images for: Functional convergence amid taxonomic variability in gut microbiome–immune checkpoint inhibitor research: a bibliometric and mechanistic synthesis
Source: Front Immunol. 2026 Jul 14;17:1883259. doi: 10.3389/fimmu.2026.1883259 (PMC13408408; doi:10.3389/fimmu.2026.1883259)

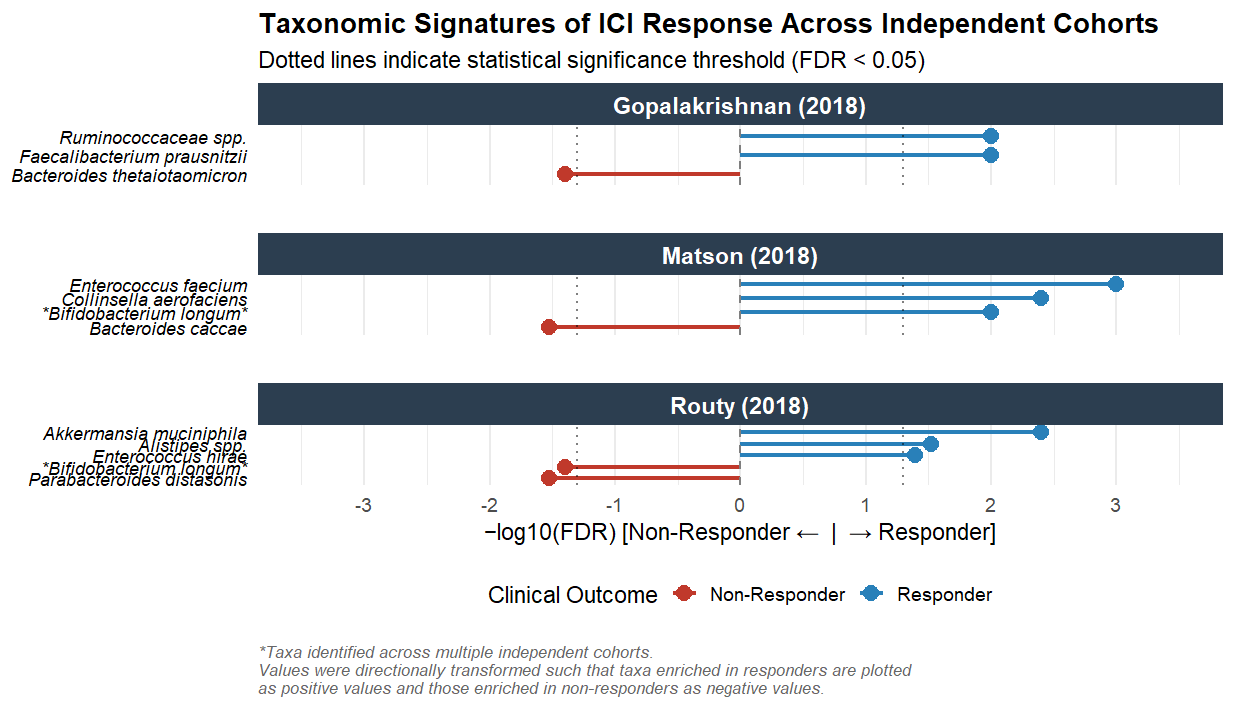

Supplement: Supplementary Figure 1 — Taxonomic signatures of immune checkpoint inhibitor response across independent clinical cohorts. Differentially abundant gut microbial taxa associated with response or non-response to immune checkpoint inhibitors (ICI) are shown across landmark clinical studies. Values represent −log10-transformed false discovery rate (FDR), with directional encoding such that taxa enriched in responders are plotted as positive values and those enriched in non-responders as negative values. Dotted vertical lines indicate the statistical significance threshold (FDR < 0.05). Taxa identified across multiple independent cohorts are indicated. [file Image1.tiff]
