# Supplementary material for: Functional convergence amid taxonomic variability in gut microbiome–immune checkpoint inhibitor research: a bibliometric and mechanistic synthesis
Source: Front Immunol. 2026 Jul 14;17:1883259. doi: 10.3389/fimmu.2026.1883259 (PMC13408408; doi:10.3389/fimmu.2026.1883259)
Supplement: Supplementary file 2 [file Table1.docx]

**Supplementary Table S1.** Characteristics of interventional clinical trials of microbiome-modulating agents combined with immune checkpoint inhibitors identified on ClinicalTrials.gov (registered March 2018 – September 2025; n = 31).

| **#** | **NCT Number** | **Indication** | **Strategy** | **Intervention** | **ICI partner** | **Phase** | **n** | **Status** |
| --- | --- | --- | --- | --- | --- | --- | --- | --- |
| 1 | NCT07191405 | Multiple solid tumors | Probiotic / LBP | L. johnsonii + chemo-immunotherapy | Tislelizumab / toripalimab | NA | 156 | Not yet recruiting |
| 2 | NCT06931808 | Locally advanced rectal cancer | FMT | FMT + chemoradio-immunotherapy | Sintilimab | Phase 4 | 20 | Enrolling by invitation |
| 3 | NCT06865521 | Advanced CRC (MSS/pMMR) | Probiotic / LBP | A. muciniphila LBP + anti–PD-1 | Anti–PD-1 | NA* | 22 | Active, not recruiting |
| 4 | NCT06486220 | Metastatic nasopharyngeal carcinoma | FMT | FMT + chemo-immunotherapy | Anti–PD-1 | Phase 3 | 96 | Not yet recruiting |
| 5 | NCT06346093 | Advanced gastric cancer | FMT | FMT capsule + chemo-immunotherapy | Anti–PD-1/PD-L1 | NA | 124 | Recruiting |
| 6 | NCT06206707 | ICI-induced colitis (melanoma, RCC) | FMT | FMT for IMC management | Anti–PD-1 (various) | NA | 20 | Recruiting |
| 7 | NCT05865730 | Advanced NSCLC and RCC | Probiotic / LBP | A. muciniphila LBP (Oncobax-AK) + anti–PD-1 | Anti–PD-1 (various) | Phase 2 | 122 | Recruiting |
| 8 | NCT05750030 | Refractory hepatocellular carcinoma | FMT | FMT + anti–PD-L1 + bevacizumab | Atezolizumab + bevacizumab | Phase 2 | 12 | Completed |
| 9 | NCT05669846 | Relapsed/refractory NSCLC | FMT | Healthy donor FMT + anti–PD-1 | Pembrolizumab | Phase 2 | 26 | Recruiting |
| 10 | NCT05533983 | Refractory solid tumors | FMT | FMT + anti–PD-1 | Nivolumab | Phase 2 | 50 | Recruiting |
| 11 | NCT05286294 | Multiple solid tumors | FMT | FMT (responder donor) + ICI | Various ICI | Phase 2 | 20 | Active, not recruiting |
| 12 | NCT05279677 | Advanced colorectal cancer | FMT | FMT + anti–PD-1 + TKI | Sintilimab + fruquintinib | Phase 2 | 30 | Unknown |
| 13 | NCT05273255 | Refractory solid tumors | FMT | FMT (responder donor) + ICI | Various ICI | NA | 18 | Completed |
| 14 | NCT05251389 | Refractory melanoma | FMT | FMT (donor comparison) + anti–PD-1 | Anti–PD-1 | Phase 1/2 | 24 | Recruiting |
| 15 | NCT05094167 | NSCLC | Probiotic / LBP | Probiotic mix (V9) + chemo-immunotherapy | Carilizumab + platinum | NA | 46 | Unknown |
| 16 | NCT05032014 | Liver cancer | Probiotic / LBP | Probiotic mix (M9) + anti–PD-1 | Anti–PD-1 | NA | 46 | Unknown |
| 17 | NCT05008861 | NSCLC | FMT | FMT capsule + chemo-immunotherapy | Anti–PD-1/PD-L1 | Phase 1 | 20 | Unknown |
| 18 | NCT05001360 | Refractory gastric cancer | FMT | FMT capsule (XBI-302) + anti–PD-1 | Nivolumab | NA | 0 | Withdrawn |
| 19 | NCT04988841 | Treatment-naive melanoma | Probiotic / LBP | Defined consortium (MaaT013) + dual ICI | Ipilimumab + nivolumab | Phase 2 | 70 | Completed |
| 20 | NCT04951583 | NSCLC and melanoma | FMT | FMT + ICI | Various ICI | Phase 2 | 45 | Active, not recruiting |
| 21 | NCT04924374 | Advanced lung cancer | FMT | FMT + anti–PD-1 | Anti–PD-1 | NA | 25 | Completed |
| 22 | NCT04758507 | Advanced renal cell carcinoma | FMT | FMT + anti–PD-1 | Anti–PD-1 | Phase 1/2 | 50 | Completed |
| 23 | NCT04729322 | Metastatic colorectal cancer | FMT | FMT + anti–PD-1 re-introduction | Pembrolizumab / nivolumab | Phase 2 | 15 | Active, not recruiting |
| 24 | NCT04577729 | Refractory melanoma | FMT | FMT + ICI re-challenge | Various ICI | NA | 5 | Terminated |
| 25 | NCT04130763 | Gastrointestinal cancer | FMT | FMT capsule + anti–PD-(L)1 | Anti–PD-1/PD-L1 | Phase 1 | 10 | Completed |
| 26 | NCT04116775 | Metastatic prostate cancer | FMT | FMT + anti–PD-1 + AR antagonist | Pembrolizumab + enzalutamide | Phase 2 | 32 | Unknown |
| 27 | NCT04038619 | ICI-induced colitis (genitourinary) | FMT | FMT for IMC management | Various ICI | Phase 1 | 40 | Recruiting |
| 28 | NCT03819296 | ICI-induced colitis (multi-cancer) | FMT | FMT for IMC management | Various ICI | Phase 1 | 800 | Recruiting |
| 29 | NCT03772899 | Advanced melanoma | FMT | Healthy donor FMT + anti–PD-1 | Pembrolizumab / nivolumab | Phase 1 | 20 | Active, not recruiting |
| 30 | NCT03686202 | Multiple solid tumors | Probiotic / LBP | Defined consortium (MET-4) + ICI | Various ICI | Phase 2/3 | 65 | Active, not recruiting |
| 31 | NCT03341143 | Advanced melanoma | FMT | Healthy donor FMT + anti–PD-1 | Pembrolizumab | Phase 2 | 20 | Completed |

*Search conducted on September 27, 2025, using the search terms (“microbiome” OR “fecal microbiota transplant”) AND (“immune checkpoint inhibitors” OR specific ICI agents). Trials are sorted by NCT number (most recent first). Strategy categories follow recognized translational frameworks; in this dataset only FMT (n = 24, 77%) and Probiotic / live biotherapeutic product (n = 7, 23%) approaches were represented, while dietary modulation, metabolite-based, and antibiotic-stewardship strategies were not present in the interventional pipeline as of the search date. Phase labels were extracted as reported in ClinicalTrials.gov; NA indicates not applicable or not assigned, often used for early-phase, exploratory, or biomarker-focused trials without a standard phase designation.* ***Abbreviations:*** *AR, androgen receptor; CRC, colorectal cancer; FMT, fecal microbiota transplantation; ICI, immune checkpoint inhibitor; IMC, immune-mediated colitis; LBP, live biotherapeutic product; MSS/pMMR, microsatellite stable / proficient mismatch repair; NA, not applicable / not assigned; NSCLC, non-small cell lung cancer; PD-L1, programmed death-ligand 1; RCC, renal cell carcinoma; TKI, tyrosine kinase inhibitor. *Described as a Phase 1 study in the trial protocol text but registered with no structured phase designation on ClinicalTrials.gov.*
