# Supplementary material for: Functional convergence amid taxonomic variability in gut microbiome–immune checkpoint inhibitor research: a bibliometric and mechanistic synthesis
Source: Front Immunol. 2026 Jul 14;17:1883259. doi: 10.3389/fimmu.2026.1883259 (PMC13408408; doi:10.3389/fimmu.2026.1883259)
Supplement: Supplementary file 3 [file Table2.docx]

# Supplementary Table S2

## Risk-of-bias appraisal of the eight studies included in the cross-cohort functional integration

**Rating key:** L = low risk; U = unclear; H = high risk; Y = yes (criterion met); UC = unclear; NA = not applicable.

## Part A. Human cohort studies appraised with the JBI Critical Appraisal Checklist for Cohort Studies

Studies appraised: Matson 2018 (Science) [ref 10]; Routy 2018 (Science) [ref 11]; Andrews 2021 (Nat Med) [ref 26]; Spencer 2021 (Science) [ref 27].

| **JBI checklist item** | **Matson 2018** | **Routy 2018** | **Andrews 2021** | **Spencer 2021** |
| --- | --- | --- | --- | --- |
| Q1. Two groups recruited from same population? | Y | Y | Y | Y |
| Q2. Exposures measured similarly between groups? | Y | Y | Y | Y |
| Q3. Exposure measured in a valid, reliable way? | Y | Y | Y | Y |
| Q4. Confounding factors identified? | Y | Y | Y | Y |
| Q5. Strategies to deal with confounding stated? | UC | Y | Y | Y |
| Q6. Groups/participants free of outcome at start? | Y | Y | Y | Y |
| Q7. Outcomes measured in valid, reliable way? | Y | Y | Y | Y |
| Q8. Follow-up time reported and sufficient? | Y | Y | Y | Y |
| Q9. Follow-up complete? If not, reasons explored? | Y | Y | Y | UC |
| Q10. Strategies to address incomplete follow-up? | UC | Y | Y | UC |
| Q11. Appropriate statistical analysis used? | Y | Y | Y | Y |
| **Overall risk-of-bias judgment** | **L** | **L** | **L** | **L** |

**Notes (Part A).** Q5 for Matson 2018 is marked unclear because the original publication did not explicitly describe confounder adjustment beyond standard demographic matching. Q9 and Q10 for Spencer 2021 are marked unclear because dietary fiber and probiotic exposure was self-reported, introducing uncertainty about completeness of exposure ascertainment. All four studies meet the threshold for low overall risk of bias under JBI criteria.

## Part B. Preclinical animal studies appraised with SYRCLE’s Risk of Bias tool

Studies appraised: Sivan 2015 (Science) [ref 12]; Mager 2020 (Science) [ref 28]; Tanoue 2019 (Nature) [ref 29]; Vétizou 2015 (Science) [ref 30].

| **SYRCLE item** | **Sivan 2015** | **Mager 2020** | **Tanoue 2019** | **Vétizou 2015** |
| --- | --- | --- | --- | --- |
| Q1. Selection bias: sequence generation adequate? | U | U | U | U |
| Q2. Selection bias: baseline characteristics similar? | L | L | L | L |
| Q3. Selection bias: allocation concealment? | U | U | U | U |
| Q4. Performance bias: random housing? | L | L | L | L |
| Q5. Performance bias: blinding of caregivers/investigators? | U | U | U | U |
| Q6. Detection bias: random outcome assessment? | L | L | L | L |
| Q7. Detection bias: blinded outcome assessment? | U | U | L | U |
| Q8. Attrition bias: incomplete outcome data addressed? | L | L | L | L |
| Q9. Reporting bias: free of selective outcome reporting? | L | L | L | L |
| Q10. Other sources of bias? | L | L | L | L |
| **Overall risk-of-bias judgment** | **L–U** | **L–U** | **L** | **L–U** |

**Notes (Part B).** Most preclinical microbiome–ICI studies pre-date routine ARRIVE / SYRCLE reporting, so unclear ratings on Q1, Q3, Q5, and Q7 are expected and reflect field-wide reporting limitations rather than specific weaknesses. Tanoue 2019 explicitly describes blinded outcome assessment, hence its low rating on Q7. Overall judgments of L–U reflect a baseline of low risk with one or two unclear domains; none of the four studies show evidence of high risk of bias on any domain.

## Part C. Study-level summary of overall risk-of-bias judgments

| **Study** | **Design** | **Tool** | **Overall RoB** | **Notes** |
| --- | --- | --- | --- | --- |
| Matson 2018 (Science) [ref 10] | Human cohort | JBI | Low | Item 5 unclear; otherwise all items met. |
| Routy 2018 (Science) [ref 11] | Human cohort | JBI | Low | All 11 JBI items met. |
| Andrews 2021 (Nat Med) [ref 26] | Human cohort | JBI | Low | All 11 JBI items met. |
| Spencer 2021 (Science) [ref 27] | Human cohort | JBI | Low | Items 9 and 10 unclear due to self-reported dietary exposure. |
| Sivan 2015 (Science) [ref 12] | Preclinical | SYRCLE | Low to unclear | Field-typical reporting gaps on allocation concealment and blinding. |
| Mager 2020 (Science) [ref 28] | Preclinical | SYRCLE | Low to unclear | Field-typical reporting gaps on allocation concealment and blinding. |
| Tanoue 2019 (Nature) [ref 29] | Preclinical | SYRCLE | Low | Explicit blinded outcome assessment reported. |
| Vétizou 2015 (Science) [ref 30] | Preclinical | SYRCLE | Low to unclear | Field-typical reporting gaps on allocation concealment and blinding. |

**Supplementary Table S2.** Risk-of-bias appraisal of the eight landmark studies included in the cross-cohort functional integration. Human cohort studies (Matson 2018, Routy 2018, Andrews 2021, Spencer 2021) were appraised using the Joanna Briggs Institute (JBI) Critical Appraisal Checklist for Cohort Studies (11 items). Preclinical animal studies (Sivan 2015, Mager 2020, Tanoue 2019, Vétizou 2015) were appraised using SYRCLE’s Risk of Bias tool for animal studies (10 items). Domain-level judgments are reported as low (L), unclear (U), or high (H) risk of bias; checklist responses are reported as yes (Y), unclear (UC), or not applicable (NA). Overall risk-of-bias judgments reflect the totality of domain ratings. Appraisal outcomes are reported to support qualitative interpretation of the included studies and were not used for quantitative weighting, pooling, or meta-analytic synthesis.
