# Supplementary material for: Functional convergence amid taxonomic variability in gut microbiome–immune checkpoint inhibitor research: a bibliometric and mechanistic synthesis
Source: Front Immunol. 2026 Jul 14;17:1883259. doi: 10.3389/fimmu.2026.1883259 (PMC13408408; doi:10.3389/fimmu.2026.1883259)
Supplement: Supplementary file 4 [file Table3.docx]

**Identification of studies via foundational microbiome–ICI literature**

Records removed *before screening*:

Duplicate records removed: (n = 0)

Records marked as ineligible by automation tools: (n = 0)

Records removed for other reasons: (n = 0)

Records identified from:

Foundational literature (n = 12)

**Identification**

Records screened

(n = 12)

Records excluded

(n = 0)

Reports sought for retrieval

(n = 12)

Reports not retrieved

(n = 0)

**Screening**

Reports assessed for eligibility

(n = 12)

Reports excluded:

Reason 1: Lacking responder/non-responder classification (n = 2)

Reason 2: Lacking taxon-level data (n = 1)

Reason 3: Lacking statistical metrics (n = 1)

Studies included in review

(n = 8)

Reports of included studies

(n = 8)

**Included**

Figure S2. PRISMA 2020 flow diagram for the cross-cohort functional integration. Twelve candidate studies were identified from the foundational microbiome–ICI literature, defined as landmark publications anchoring the bibliometric co-citation network and key narrative reviews of the field. Studies were screened and assessed against three eligibility criteria: clearly defined responder and non-responder classifications, taxon-level differential abundance data, and associated statistical metrics. Four studies were excluded at the eligibility stage; the final synthesis included eight peer-reviewed studies (four human observational cohorts: Matson 2018, Routy 2018, Andrews 2021, Spencer 2021; four preclinical animal studies: Sivan 2015, Mager 2020, Tanoue 2019, Vétizou 2015).
